# Supplementary material for: Inhibition of DDR1‐BCR signalling by nilotinib as a new therapeutic strategy for metastatic colorectal cancer
Source: EMBO Mol Med. 2018 Feb 9;10(4):e7918. doi: 10.15252/emmm.201707918 (PMC5887546; doi:10.15252/emmm.201707918)
Supplement: Supplementary file 1 — Appendix [file EMMM-10-e7918-s001.pdf]

Appendix

Inhibition of DDR1-BCR signalling by nilotinib as a new therapeutic strategy for metastatic colorectal cancer

Table of Contents:

Appendix Table S1. Univariate and multivariate analysis. p2

Appendix Table S2. Statistical analysis information for main figures. p3

Appendix Tables S3. Statistical analysis information for EV figures. p4

Appendix Figure S1. Luciferase signal as a surrogate marker of liver tumour burden from Figure 3F. p5

Appendix Figure S2. No significant difference in the expression level of DDR1 between the four CMS subtypes. p6

Appendix Figure S3. DDR1 expression (A, B) and nilotinib treatment (C) poorly affect CRC cell growth in standard conditions. p7

Appendix Table S1. Univariate and multivariate analysis.

Univariate analyse			PFS			OS	
	**N=143(%)**	**HR**	**95%CI**	**p***	**HR**	**95%CI**	**p***
**DDR1 (210749_x_1)**				**0.006**			**0.062**
< 10.6	72(50.3)	1			1		
≥ 10.6	71(49.7)	1.62	[1.15 ; 2.30]		1.44	[0.98 ; 2.10]	
**WHO performance status**				**0.022**			0.262
0	60(43.8)	1			1		
≥1	77(56.2)	1.52	[1.06 ; 2.19]		1.25	[0.84 ; 1.85]	
Missing	6						
**Tumor location**				0.329			**0.010**
Right	35(24.7)	1			1		
Left	104(73.2)	0.83	[0.56 ; 1.23]		0.65	[0.42 ; 1.01]	
Transverse	3(2.1)	1.88	[0.57 ; 6.22]		4.81	[1.38 ; 16.7]	
Missing	1						
**Grade**				**0.077**			**0.006**
Well/moderately diff	122(91.0)	1			1		
Poorly diff / undifferentiated	12(9.0)	1.79	[0.98 ; 3.27]		2.61	[1.42 ; 4.82]	
Missing	9						
**Metastase**				0.775			0.421
Synchronous	121(84.6)	1			1		
Métachronous	22(15.4)	1.07	[0.67 ; 1.73]		0.80	[0.46 ; 1.39]	
**Number of metastatic sites**				**0.003**			**0.034**
1	82 (57.3)	1				1	
>1	61 (42.7)	1.71	[1.21 ; 2.43]			1.51	[1.03 ; 2.
**Status**				0.670			**0.027**
MSI	4(10.5)	1				1	
MSS	34(89.5)	0.79	[0.27 ; 2.27]			0.23	[0.08 ; 0.
Missing	105						
**BRAF**				0.668			0.458
WT	30(93.8)	1			1		
Mutated	2(6.2)	1.39	[0.33 ; 5.97]		0.49	[0.06 ; 3.82]	
Missing	111						
**KRAS**				0.336			0.240
WT	18(54.5)	1			1		
Mutated	15(45.5)	1.43	[0.69 ; 2.94]		1.62	[0.73 ; 3.59]	
Missing	110						
**CMS**				**0.201**			**0.030**
CMS1	15(14.4)	1			1		
CMS2	29(27.9)	0.84	[0.44 ; 1.61]		0.38	[0.19 ; 0.77]	
CMS3	24(23.1)	0.68	[0.34 ; 1.36]		0.33	[0.16 ; 0.70]	
CMS4	36(34.6)	0.54	[0.29 ; 1.02]		0.36	[0.18 ; 0.70]	
Missing	39						

Multivariate analyse		PFS			OS	
	**HR**	**95%CI**	**p***	**HR**	**95%CI**	**p***
**DDR1 (210749_x_1)**			**0.003**			
< 10.6	1					
≥ 10.6	1.76	[1.21 ; 2.55]				
**Tumor location**						**0.00**
Right				1		
Left				0.54	[0.30 ; 0.97]	
Transverse				8.31	[2.14 ; 32.3]	
**Grade**						**0.00**
Well/moderately diff				1		
Poorly diff / undifferentiated				4.28	[2.07 ; 8.85]	
**Number of metastatic sites**			**0.010**			
1	1					
>1	1.64	[1.13 ; 2.37]				
**CMS**						**0.00**
CMS1				1		
CMS2				0.28	[0.12; 0.62]	
CMS3				0.27	[0.11; 0.63]	
CMS4				0.21	[0.10; 0.46]	

Appendix Table S2. Statistical analysis information for main figures.

Figure	Panel	Experiment	n-value	p-value	Significativity
1	B	nilotinib vs DMSO	17	0,0269	*
	D	nilotinib vs DMSO	11	0,0447	*
2	B	HCT116 shRNA DDR1.1 vs shRNA Ctrl	4	0,0022	**
		HCT116 shRNA DDR1.2 vs shRNA Ctrl	4	0,0001	***
		HT29 shRNA DDR1.1 vs shRNA Ctrl	4	0,0008	***
		HT29 shRNA DDR1.2 vs shRNA Ctrl	4	0,0005	***
2	C	HCT116 shRNA DDR1.1 vs shRNA Ctrl	7	<0,0001	***
		HCT116 shRNA DDR1.2 vs shRNA Ctrl	7	0,0001	***
2	D	shRNA DDR1 vs shRNA Ctrl (metastatic index)	12	0,0082	**
		shRNA DDR1 vs shRNA Ctrl (ctDNA)	12	0,0461	*
2	F	SW620 DDR1 vs mock	3	0,0325	*
		HT29 DDR1 vs mock	3	0,05	*
2	G	DDR1 vs mock (metastatic index)	20 ; 12	0,0356	*
		DDR1 vs mock (ctDNA)	20 ; 12	0,0432	*
3	C	shCtrl nilotinib vs DMSO	4	0,0025	**
		shDDR1 + WT nilotinib vs DMSO	4	0,0076	**
3	D	shDDR1+DDR1T701I vs shDDR1+DDR1 WT	5	0,0178	*
3	E	SW620 mock DMSO vs SW620 DDR1 DMSO	3	0,0325	*
		SW620 DDR1 nilotinib vs SW620 DDR1 DMSO	3	0,0087	**
3	F	nilotinib vs DMSO (metastatic index)	14 ; 21	0,0425	*
		nilotinib vs DMSO (ctDNA)	14 ; 21	0,0016	**
5	F	shRNA BCR vs shRNA Ctrl (invasion)	3	0,0076	**
		shRNA BCR vs shRNA Ctrl (3D migration)	7	0,0008	***
5	H	HCT116 shBCR+BCR WT vs shBCR	4	0,0389	*
		HCT116 shBCR vs shCtrl	4	0,0028	**
		SW620 DDR1 shBCR+BCR WT vs shBCR	4	0,0472	*
		SW620 DDR1 shBCR vs shCtrl	4	0,0272	*
6	A	b-catenin vs mock	6	0,0022	**
		DDR1 vs mock	6	0,0022	**
6	B	b-catenin vs mock	6	0,016	*
		DDR1 vs mock	6	0,0022	**
6	C	DDR1 vs mock (Fra1 mRNA)	6	0,0385	*
		DDR1+nilotinib vs DDR1 (Fra1 mRNA)	6	0,04	*
		DDR1 KD vs DDR1 (Fra1 mRNA)	6	0,1447	ns
		DDR1 vs mock (Myc mRNA)	6	0,0244	*
		DDR1+nilotinib vs DDR1 (Myc mRNA)	6	0,0053	**
		DDR1 KD vs DDR1 (Myc mRNA)	6	0,4559	ns
		DDR1 vs mock (Jun mRNA)	6	0,0005	***
		DDR1+nilotinib vs DDR1 (Jun mRNA)	6	0,0002	***
		DDR1 KD vs DDR1 (Jun mRNA)	6	0,0256	*
6	D	shRNA DDR1 vs shRNA Ctrl (Fra1 mRNA)	6	0,0043	**
		shRNA DDR1 vs shRNA Ctrl (Myc mRNA)	6	0,005	**
		shRNA DDR1 vs shRNA Ctrl (Jun mRNA)	6	0,043	*
6	E	BCR WT -COL vs +COL	3	0,03	*
		BCR WT +COL vs +Nilo	3	0,0005	***
		BCR WT +COL vs +Wnt3a	3	0,5272	ns
		BCR Y177F -COL vs +COL	3	0,0221	*
6	F	nilotinib vs DMSO	20	<0,0001	***
6	G	SW620 DDR1 DMSO vs SW620 mock DMSO	3	0,0346	*
		SW620 DDR1 IWR-1-endo vs DMSO	3	0,0347	*
7	B	Metastatic nodules vs primary tumours	18	0,0314	*
7	D	CPP19 nilotinib 300nM vs Ctrl	3	0,0383	*
		CPP30 nilotinib 100nM vs Ctrl	3	0,0297	*
		CPP30 nilotinib 300nM vs Ctrl	3	0,0157	*
		CPP19 siRNA DDR1 vs Ctrl	6	0,0028	**
		CPP30 siRNA DDR1 vs Ctrl	6	0,0028	**
7	G	CTC44 nilotinib 100nM vs Ctrl	4	0,0319	*
		CTC44 nilotinib 300nM vs Ctrl	4	0,0097	**
		CTC45 nilotinib 300nM vs Ctrl	4	0,0097	**
7	H	siRNA DDR1 vs Ctrl	4	0,002	**
7	I	nilotinib vs DMSO	10	0,0089	**

Appendix Table S3. Statistical analysis information for EV figures.

Figure	Panel	Experiment	n-value	p-value	Significativity
EV4	A	mRNA CD44	3	<0,0001	***
		mRNA CCND1	3	0,0132	*
		mRNA LGR5	6	0,0002	***
		mRNA AXIN2	4	0,0193	*
		mRNA ASCL2	8	0,4228	ns
		mRNA SLC12A2	8	0,7759	ns
	B	mock-COL vs mock+COL	3	0,0221	*
		DDR1-COL vs DDR1+COL	3	0,0013	**
		mock-COL vs DDR1-COL	3	0,0001	***
		mock+COL vs DDR1+COL	3	<0,0001	***
	C	mock vs DDR1	9	0,0013	**
EV5	B	HCT116 shBCR vs shCtrl	4	0,0211	*
		SW620 shBCR vs shCtrl	4	0,0211	*

Appendix Figures

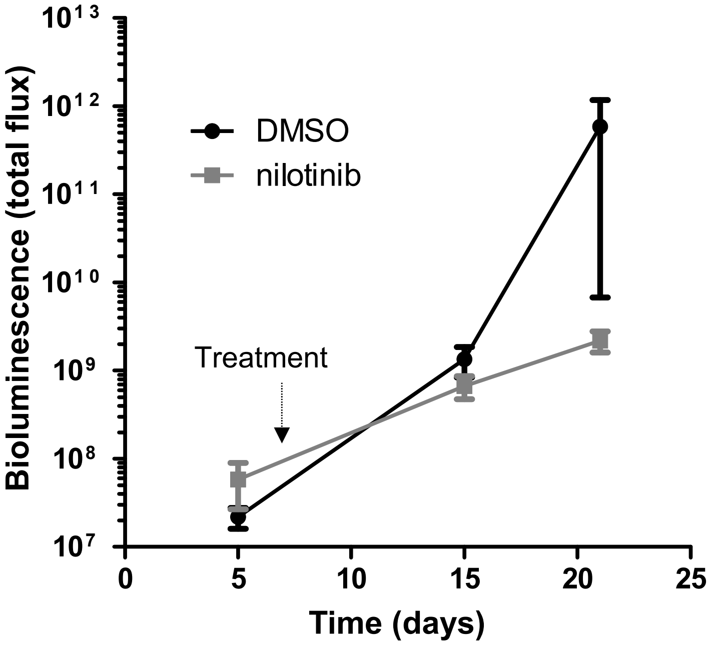

Appendix Figure S1. Luciferase signal as a surrogate marker of liver tumour burden from Figure 3F.

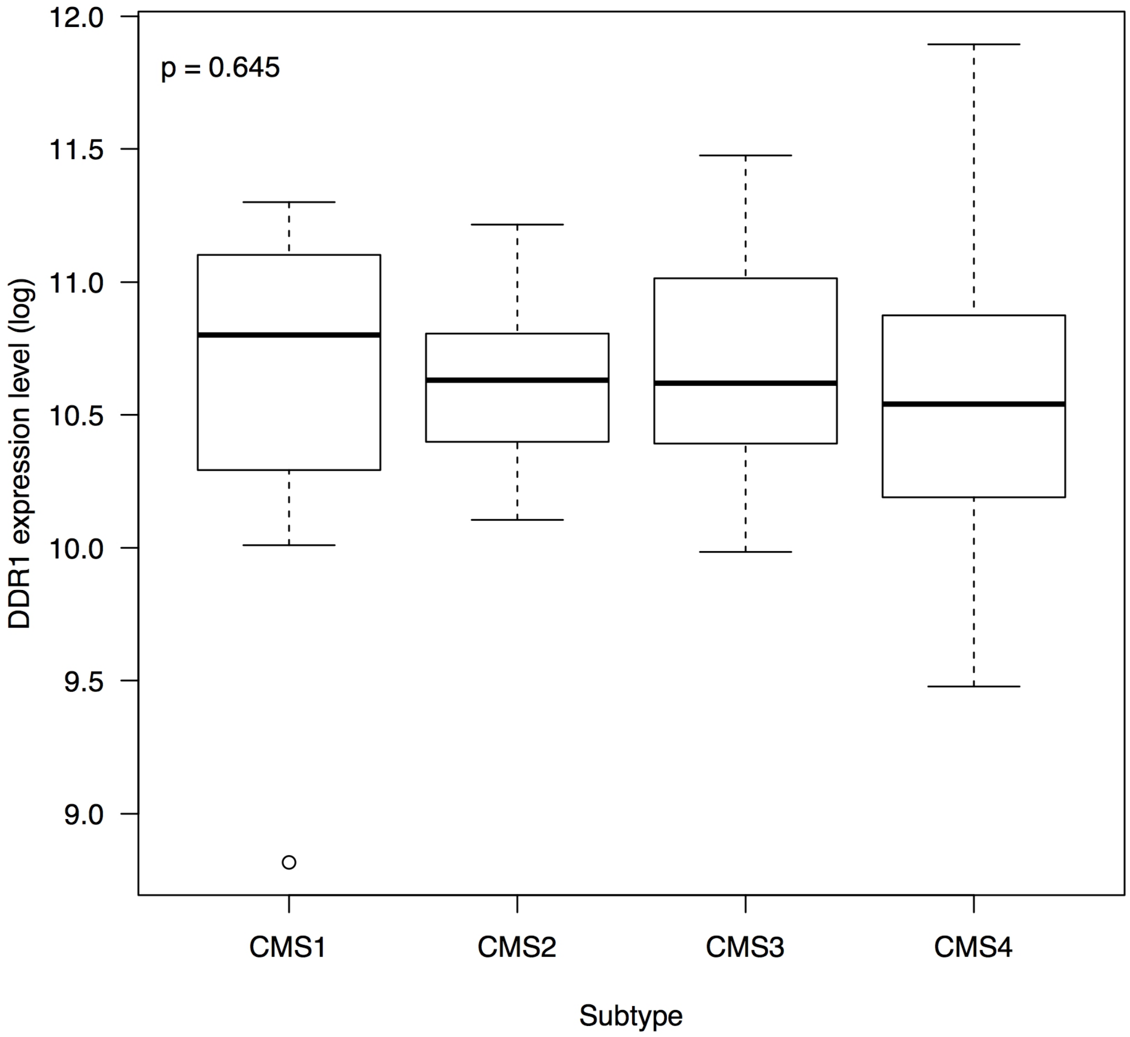

Appendix Figure S2. No significant difference in the expression of DDR1 between the four CMS subtypes.

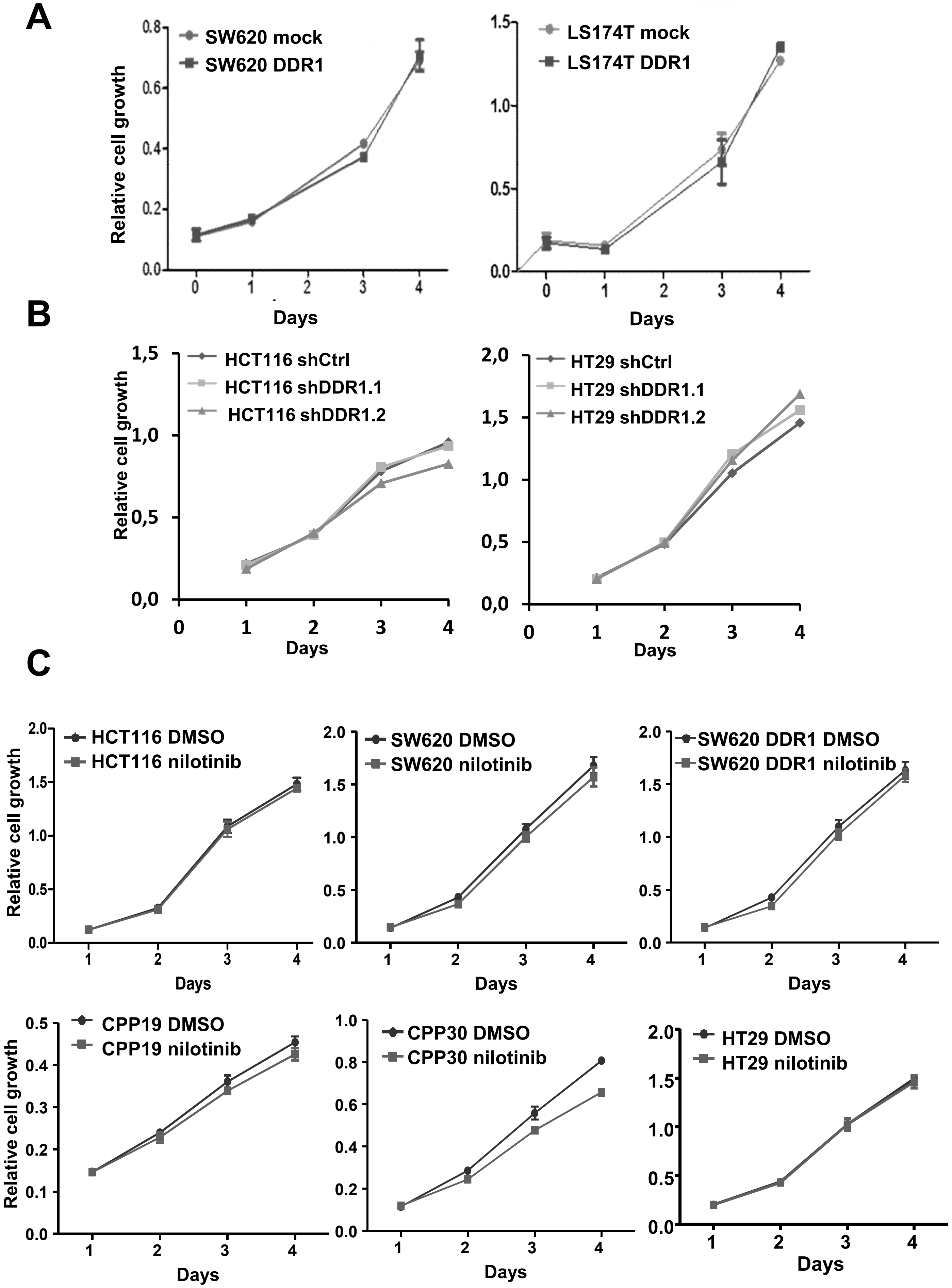

Appendix Figure S3. DDR1 expression (A,B) and nilotinib treatment (C) poorly affect CRC cell growth in standard conditions.
