# Supplementary material for: Inhibition of DDR1‐BCR signalling by nilotinib as a new therapeutic strategy for metastatic colorectal cancer
Source: EMBO Mol Med. 2018 Feb 9;10(4):e7918. doi: 10.15252/emmm.201707918 (PMC5887546; doi:10.15252/emmm.201707918)
Supplement: Supplementary file 2 — Expanded View Figures PDF [file EMMM-10-e7918-s002.pdf]

Expanded View Figures

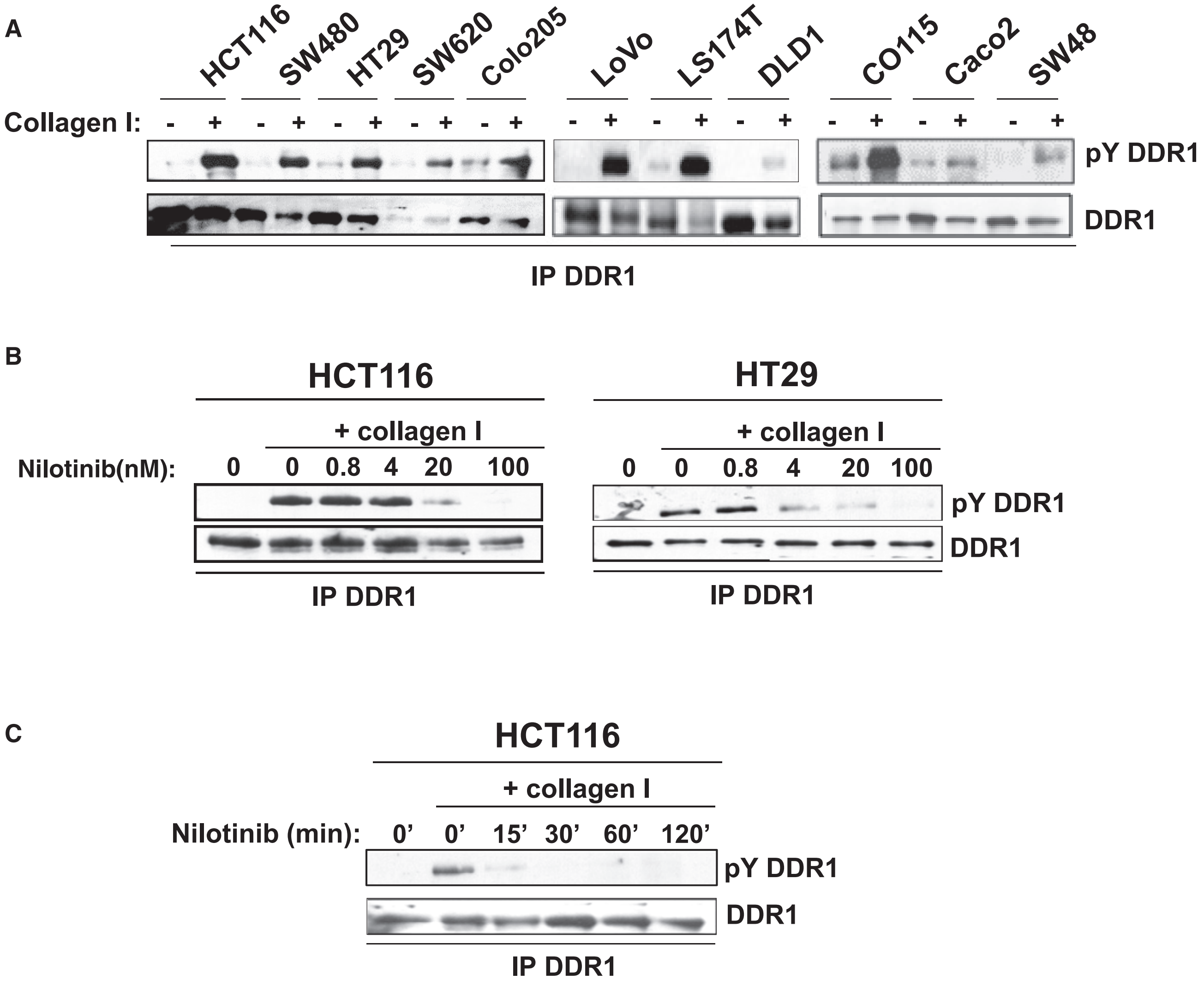

Figure EV1. Nilotinib inhibits collagen-mediated DDR1 phosphorylation.

A Collagen I induces DDR1 tyrosine phosphorylation (pY DDR1).

B, C Nilotinib inhibits DDR1 tyrosine phosphorylation. DDR1 tyrosine phosphorylation level was assessed by Western blotting in protein lysates from the indicated CRC cell lines after stimulation with 40 μg/ml collagen I for 18 h and incubation with the indicated concentrations of nilotinib and for the indicated times with 100 nM of nilotinib.

© 2018 The Authors

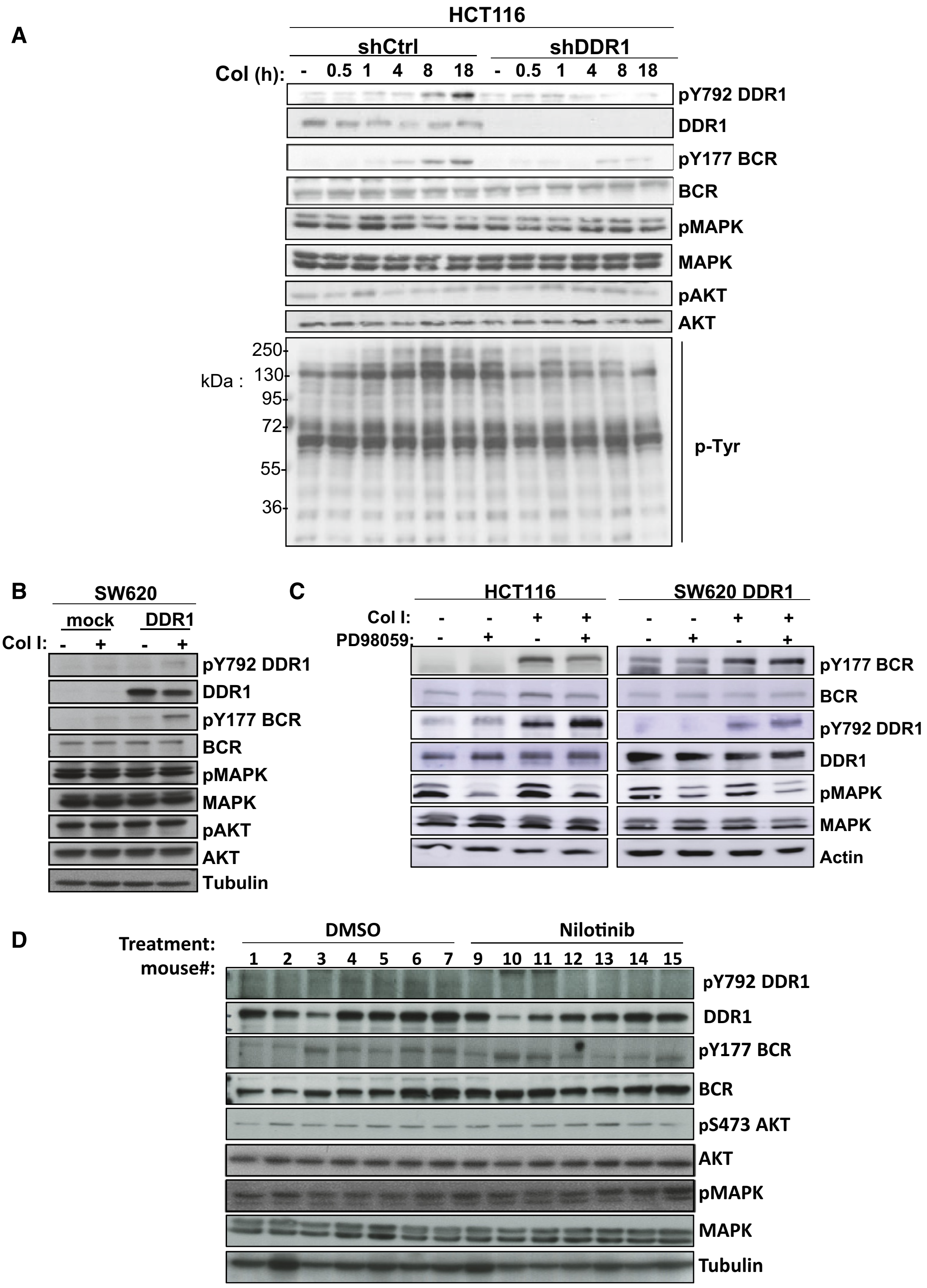

Figure EV2. RAS-independent nature of DDR1 signalling.

A, B Depletion of DDR1 abolishes (A) and overexpression of DDR1 induces (B) BCR but not MAPK or AKT phosphorylation. Tyrosine phosphorylation (pTyr) and phosphorylation levels of DDR1, BCR, MAPK and AKT were assessed by Western blotting in protein lysates from the indicated CRC cell lines stimulated or not with 40 μg/ml collagen I (Col I) for the indicated times.

C MAPK inhibition (treatment with 10 μM of PD98059 during 24 h) does not impact on DDR1 signalling.

D DDR1 signalling in CRC liver metastases. DDR1, BCR, MAPK and AKT activities from protein lysates of liver metastases described in Fig 3F.

© 2018 The Authors

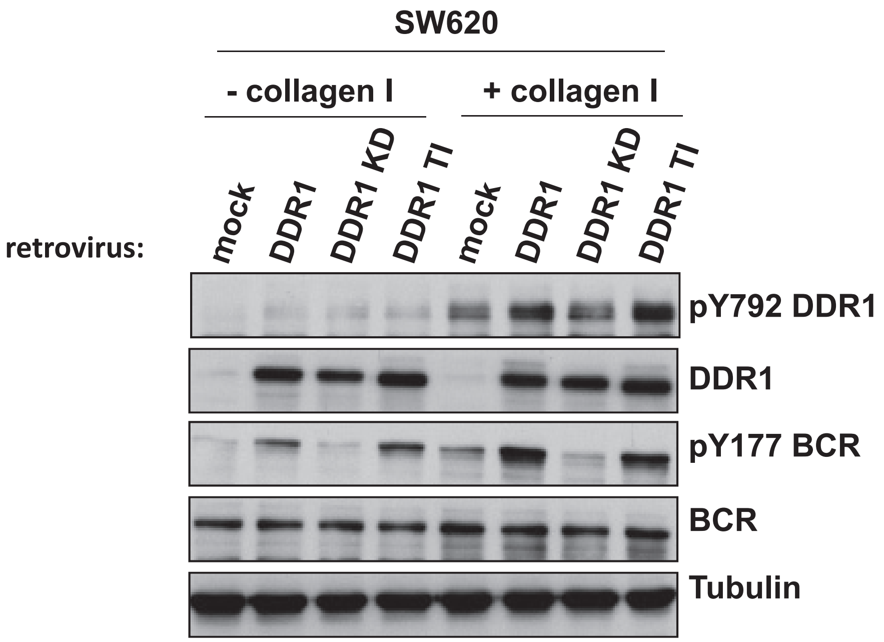

Figure EV3. BCR phosphorylation is DDR1 kinase-dependent.

Western blotting to assess pY792 DDR1 and pY177 BCR levels in SW620 cells that overexpress wild-type DDR1 (DDR1), a kinase-dead DDR1 mutant (DDR1 KD) or the gatekeeper mutant DDR1 (DDR1 TI) or not (mock), after stimulation or not with 40 μg/ml collagen I for 18 h.

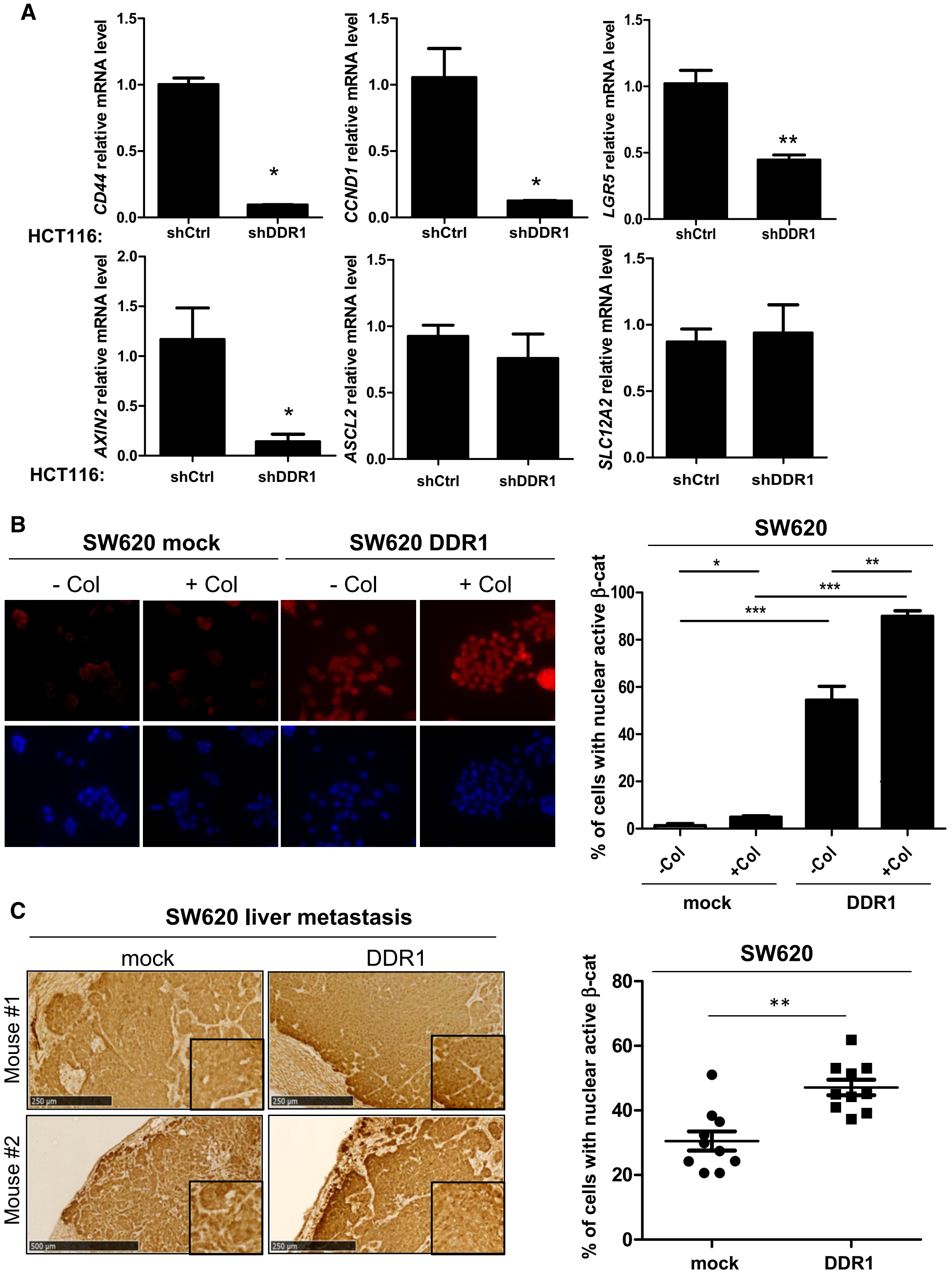

Figure EV4. DDR1 regulates β-catenin signalling in CRC cells.

A Relative transcript level of the indicated genes in HCT116 cells expressing DDR1 or Ctrl shRNA (mean ± SEM; $n = 2$; *$P < 0.05$ **$P < 0.01$; Mann–Whitney test).

B, C Representative images (left panels) and quantifications (right panels) of active β-catenin nuclear staining in control or DDR1-overexpressing SW620 cells (B) and derived liver metastasis (C). Are shown, respectively, means ± SEM; $n = 2$ (B) and $n = 5$ tumours per group with 2 fields per tumour (C); *$P < 0.05$ **$P < 0.01$ ***$P < 0.001$ Mann–Whitney test. Scale bars: 250 μm.

© 2018 The Authors

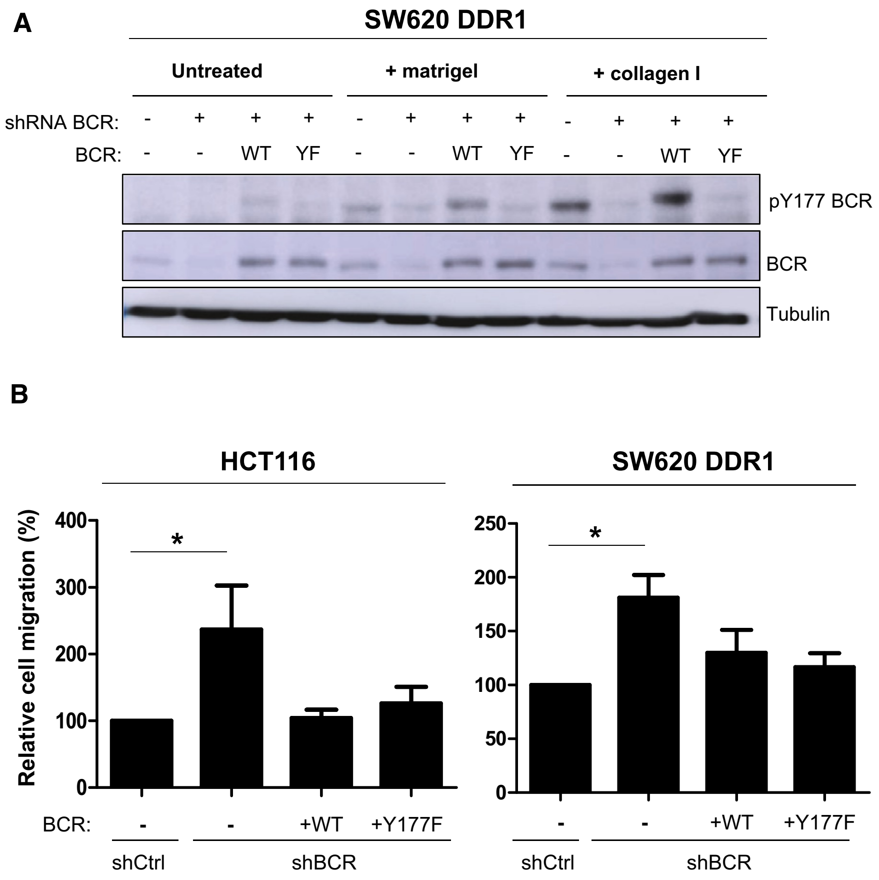

Figure EV5. Anti-migratory activity of BCR.

A BCR and pTyr177-BCR levels were assessed in CRC cells expressing both indicated shRNAs and BCR constructs (wild type, WT; Y177F, YF) and stimulated or not with collagen I (40 mg/ml for 18 h).

B Migration assays in Boyden chambers of the indicated cell lines (mean ± SEM; n = 5; *P < 0.05 Student's t-test).

© 2018 The Authors
