# Supplementary figures and images for: Inhibition of DDR1‐BCR signalling by nilotinib as a new therapeutic strategy for metastatic colorectal cancer
Source: EMBO Mol Med. 2018 Feb 9;10(4):e7918. doi: 10.15252/emmm.201707918 (PMC5887546; doi:10.15252/emmm.201707918)

B

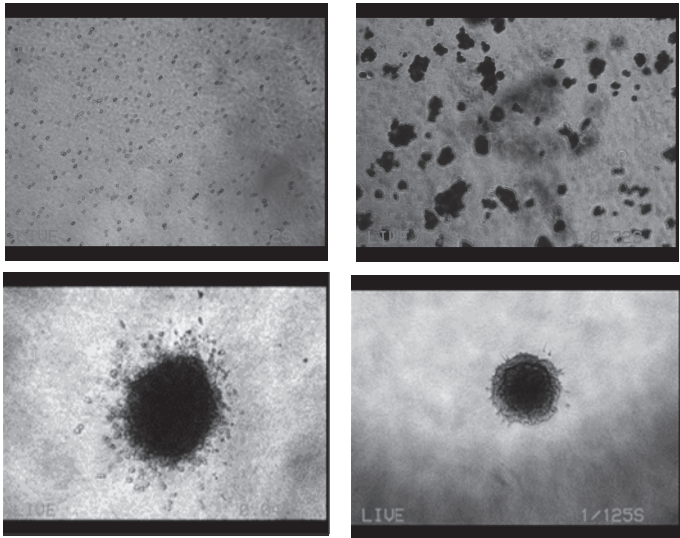

D

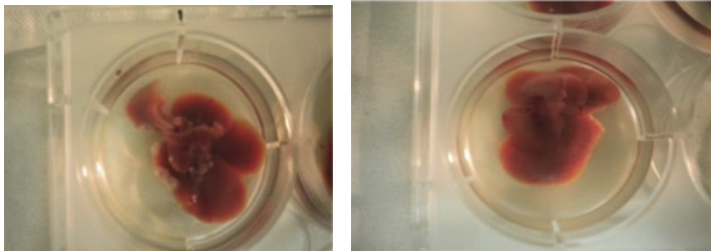

Supplement: Supplementary file 6 — Source Data for Figure 1 [file EMMM-10-e7918-s005.pdf]

Figure 2

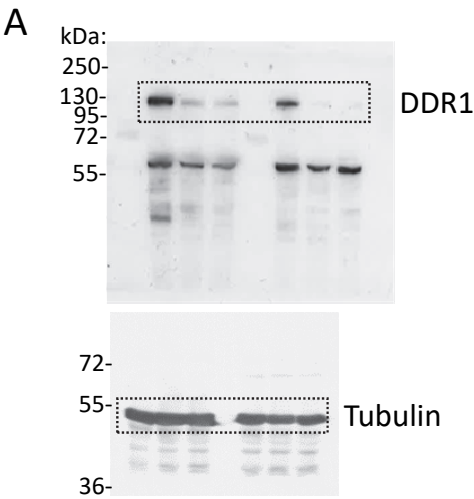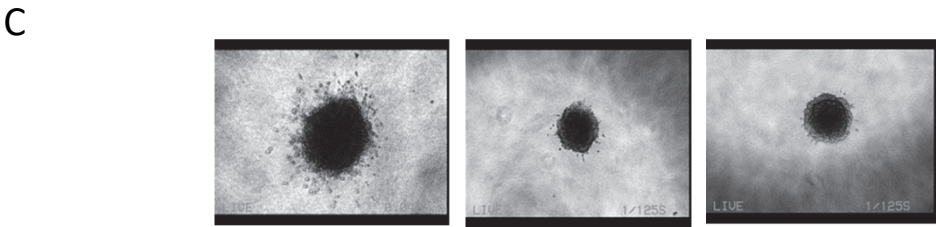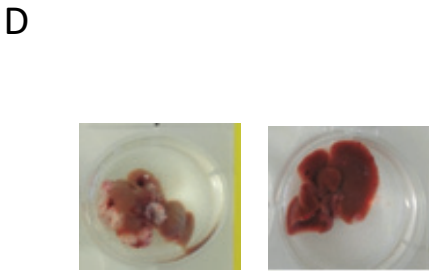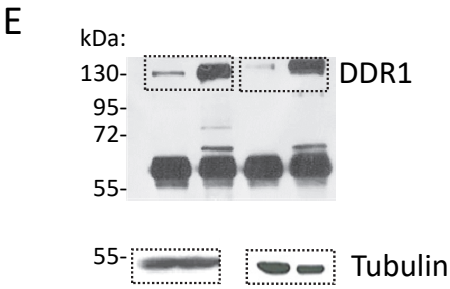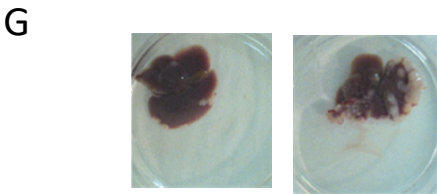

Supplement: Supplementary file 7 — Source Data for Figure 2 [file EMMM-10-e7918-s006.pdf]

Figure 3

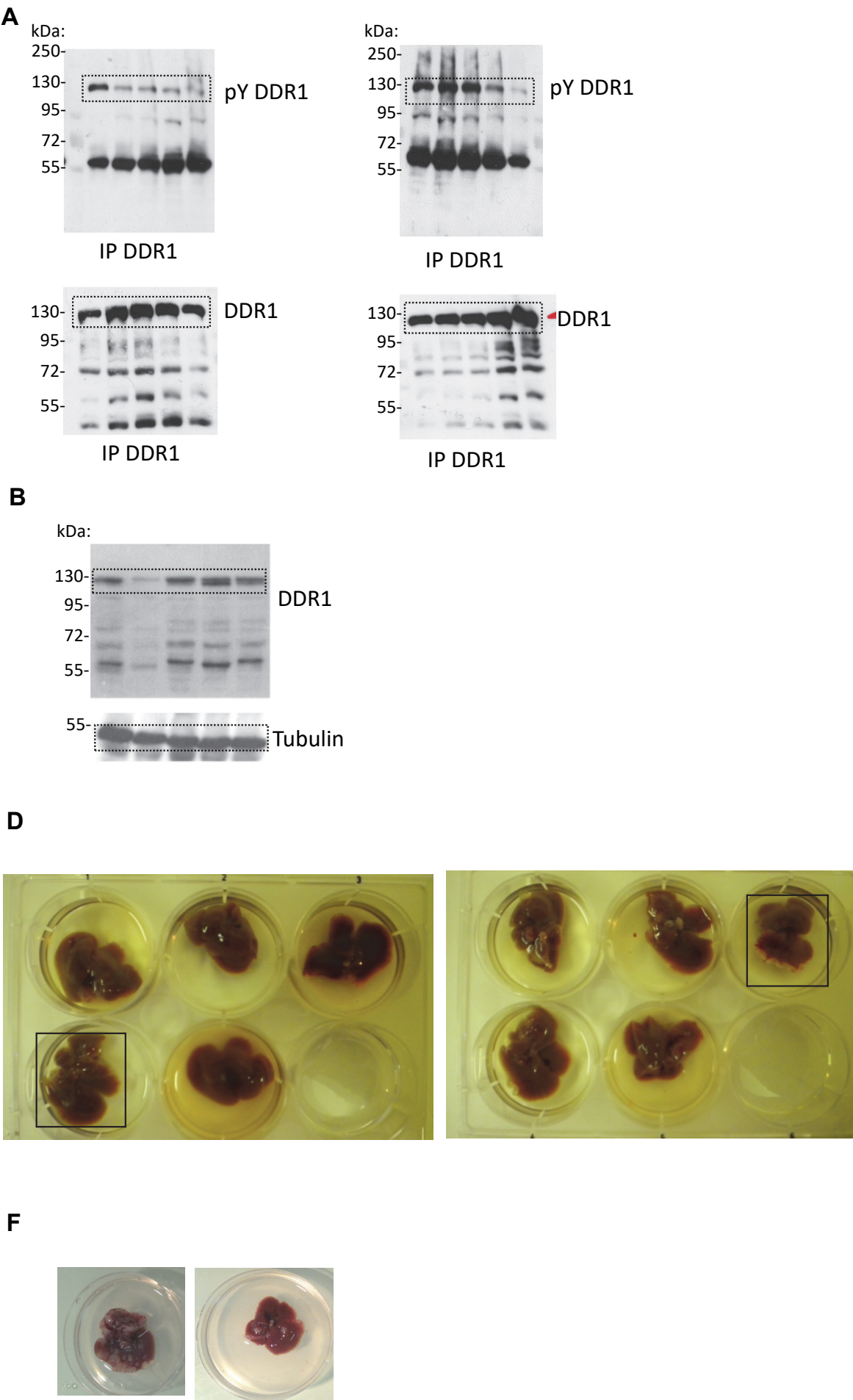

Supplement: Supplementary file 8 — Source Data for Figure 3 [file EMMM-10-e7918-s007.pdf]

Figure 4

B

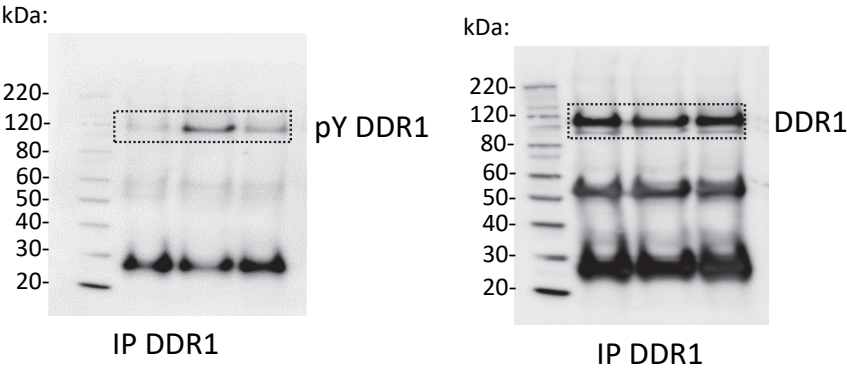

D

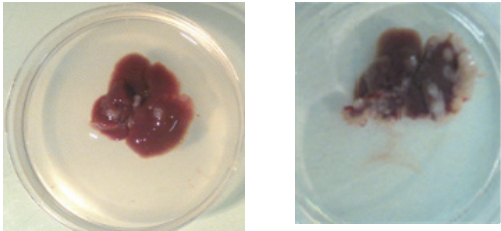

Supplement: Supplementary file 9 — Source Data for Figure 4 [file EMMM-10-e7918-s008.pdf]

Figure 5

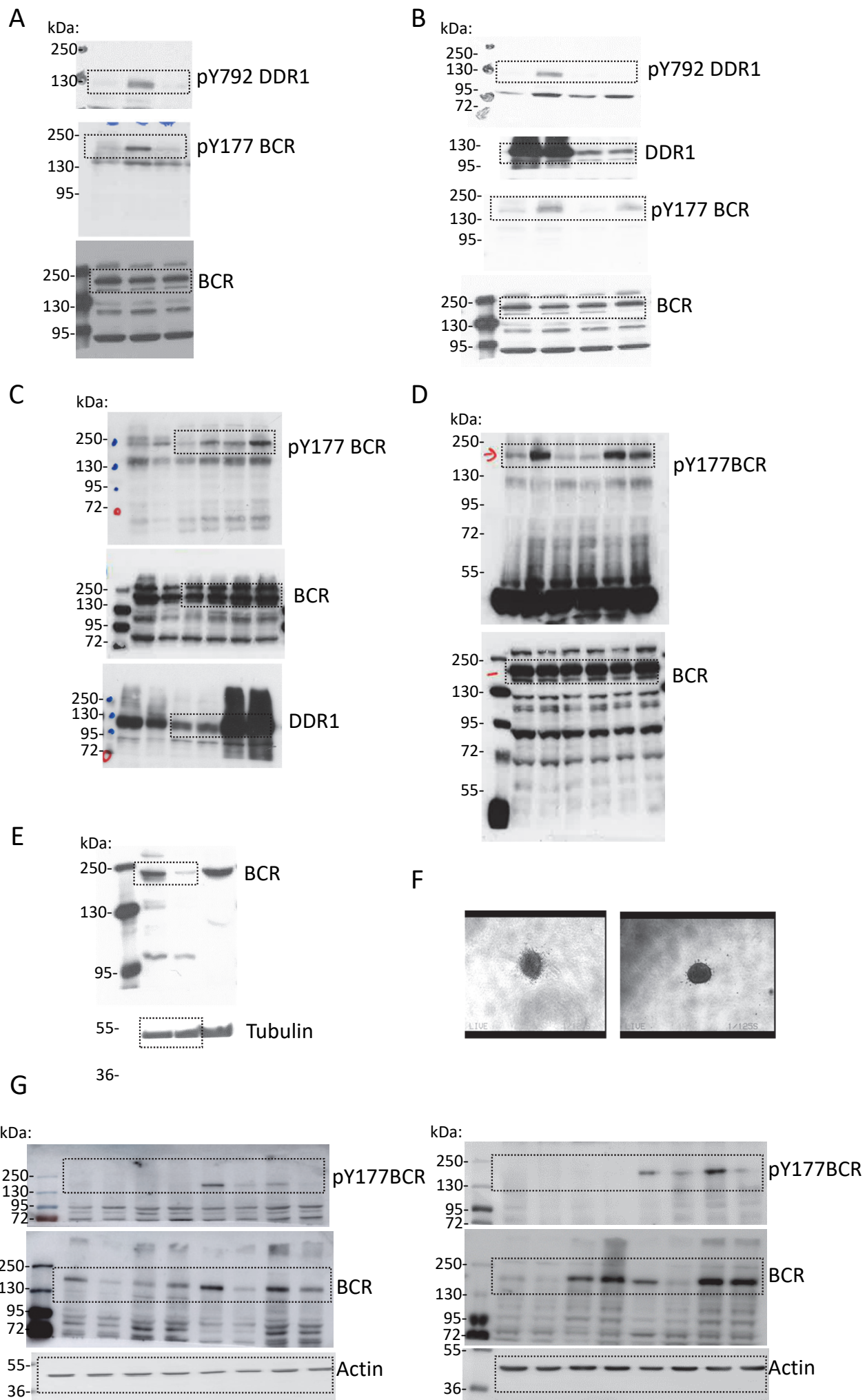

Supplement: Supplementary file 10 — Source Data for Figure 5 [file EMMM-10-e7918-s009.pdf]

Figure 6

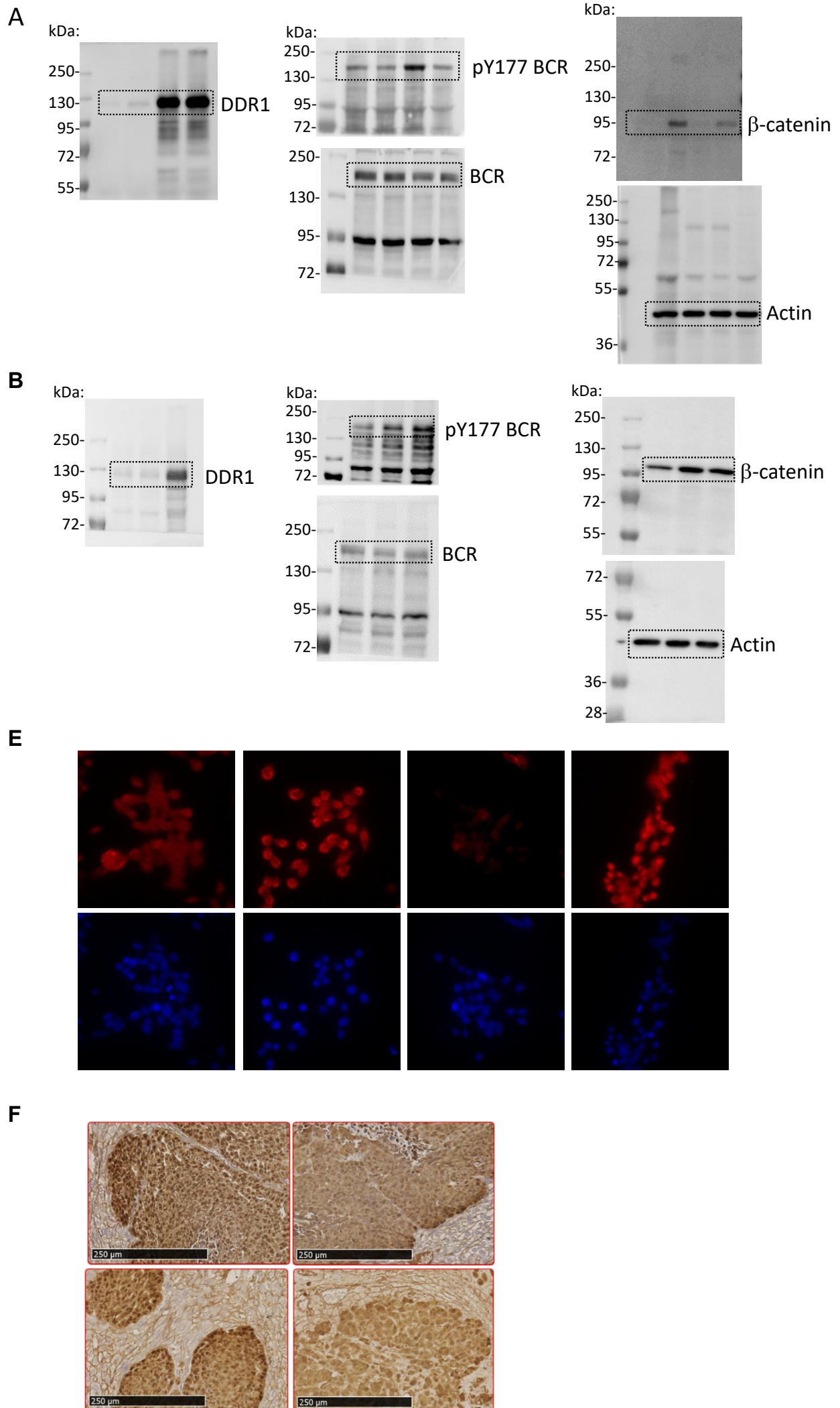

Supplement: Supplementary file 11 — Source Data for Figure 6 [file EMMM-10-e7918-s010.pdf]

**Figure 7**

**B**

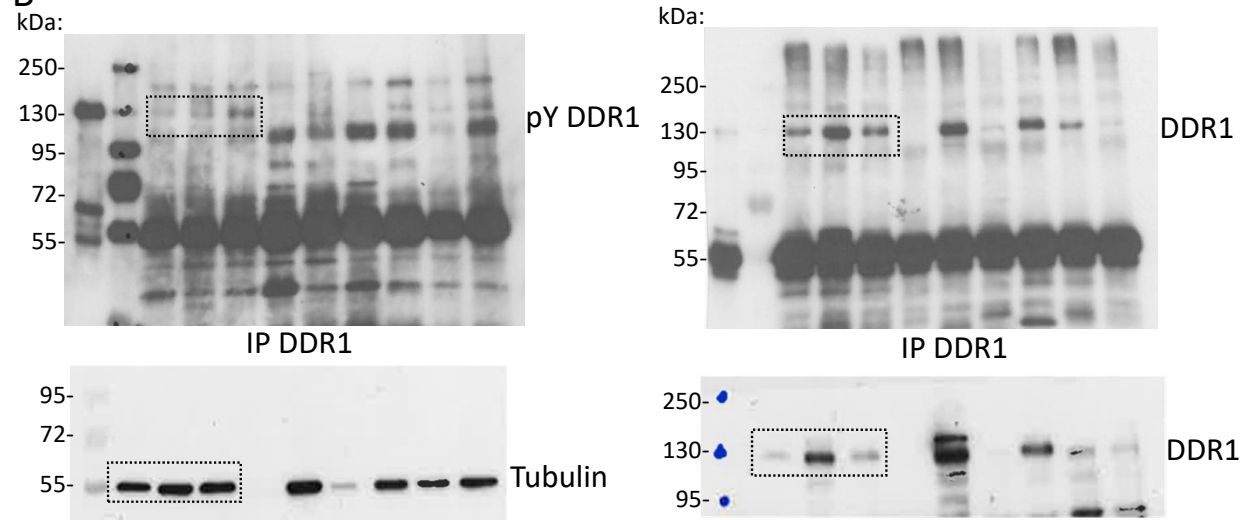

**C**

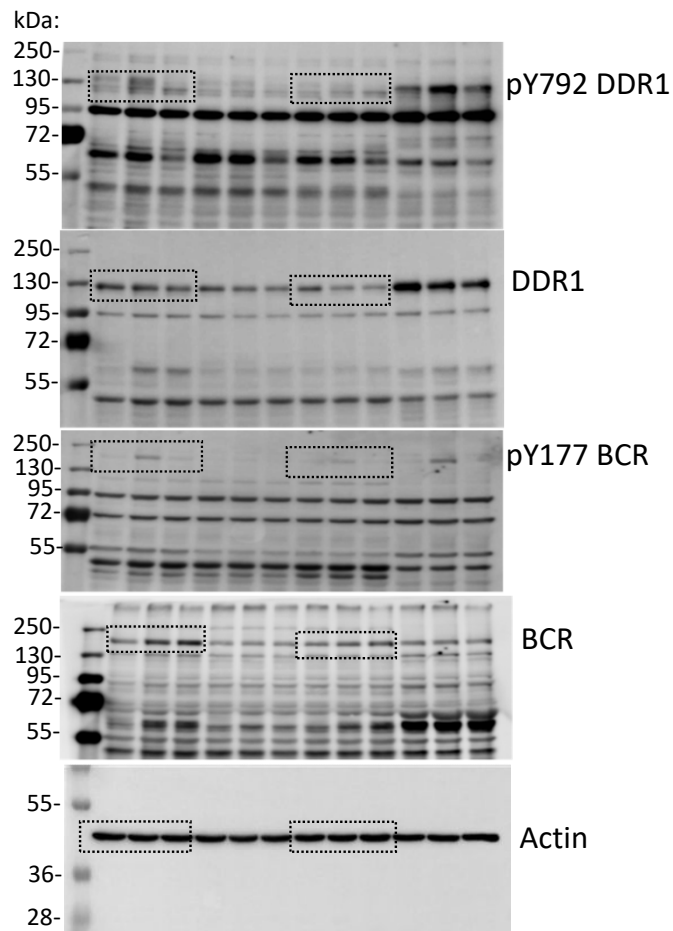

**E**

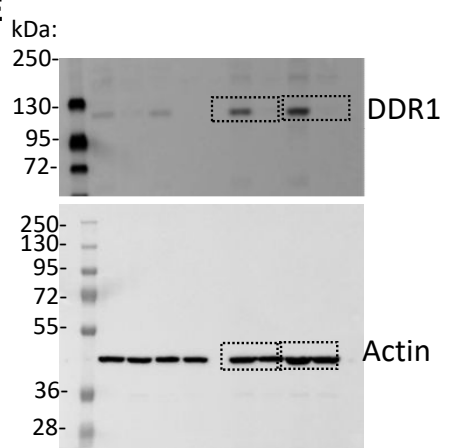

**H**

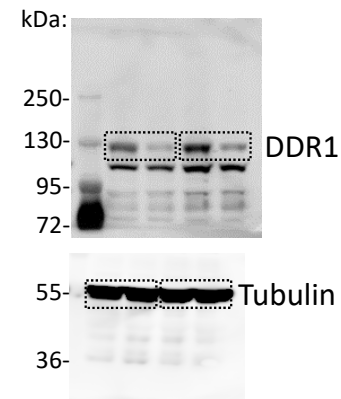

**F**

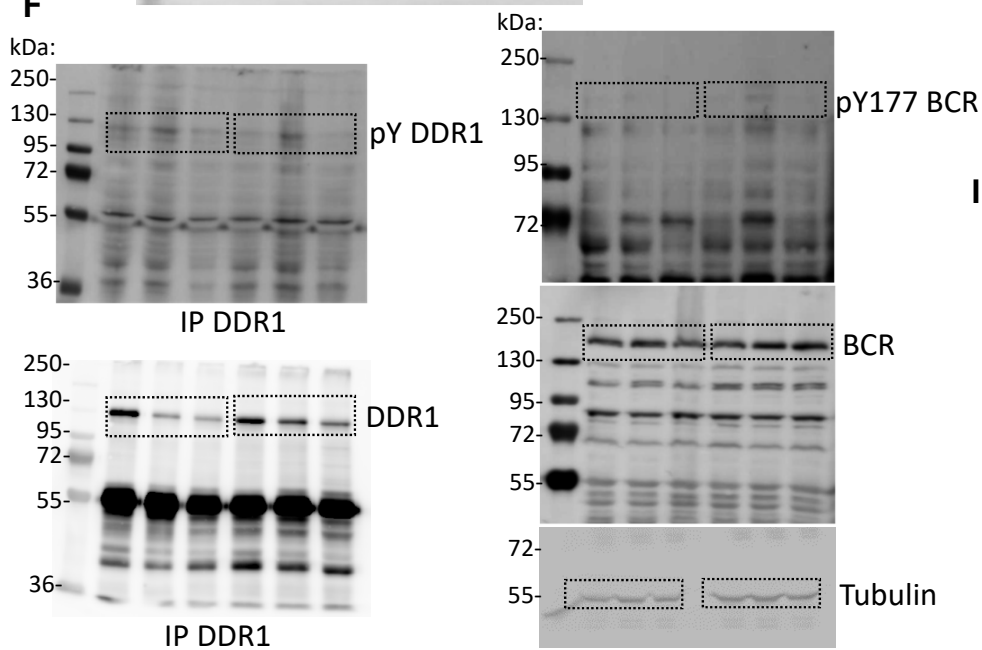

**I**

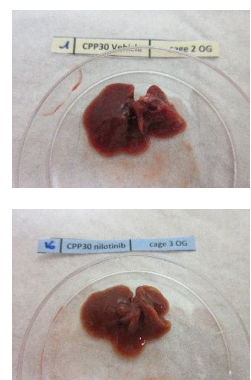

Supplement: Supplementary file 12 — Source Data for Figure 7 [file EMMM-10-e7918-s011.pdf]
